# Comparison of Antioxidants: The Limited Correlation between Various Assays of Antioxidant Activity

**DOI:** 10.3390/molecules25143244

**Published:** 2020-07-17

**Authors:** Katarzyna Naparło, Mirosław Soszyński, Grzegorz Bartosz, Izabela Sadowska-Bartosz

**Affiliations:** 1 Department of Analytical Biochemistry, Institute of Food Technology and Nutrition, College of Natural Sciences, Rzeszow University, 35-601 Rzeszow, Poland; katarzyna.naparlo@gmail.com; 2 Department of Molecular Biophysics, Faculty of Biology and Environmental Protection, University of Lodz, 90-236 Lodz, Poland; miroslaw.soszynski@biol.uni.lodz.pl; 3 Department of Bioenergetics, Food Analysis and Microbiology, Institute of Food Technology and Nutrition, College of Natural Sciences, Rzeszow University, 35-601 Rzeszow, Poland; gbartosz@ur.edu.pl

**Keywords:** antioxidant, lipid peroxidation, AAPH, hemolysis, glutathione, reactive oxygen species, hemoglobin

## Abstract

The inhibitory effects a range of synthetic and natural antioxidants on lipid peroxidation of egg yolk and erythrocyte membranes induced by a free radical generator 2,2′-azobis(2-amidinopropane) dihydrochloride (AAPH) was compared, with significant differences being found between both systems. When the protection by selected antioxidants against the effects of AAPH on erythrocytes (hemolysis, oxidation of hemoglobin and glutathione (GSH) and generation of reactive oxygen species (ROS)) was studied, most antioxidants were protective, but in some tests (oxidation of hemoglobin and GSH) some acted as prooxidants, inducing oxidation in the absence of AAPH and enhancing the AAPH-induced oxidation. These results demonstrate a diversified action of antioxidants in different systems and point to a need for careful extrapolation of any conclusions drawn from one parameter or experimental system to another.

## 1. Introduction

Antioxidants are indispensable for the proper functioning of aerobic organisms and are important in the food industry for the preservation of oxidizable products. They are also used in cosmetics, are believed to be prophylactic agents against various diseases as well as proposed to be potential therapeutics [1]. For food preservation, efficient synthetic antioxidants are usually employed, nevertheless there are concerns about their possible adverse effects [2,3,4]. Administration of antioxidants for the prevention and possible treatment of diseases imposes additional requirements such as bioavailability, lack of toxicity and the ability to penetrate the blood-brain barrier in the case of antioxidants aimed to ameliorate neurodegenerative diseases [5,6]. Often, rankings of antioxidants are created for different purposes based on their efficiency in particular systems. However, procedures proposed to determine antioxidant activity often yield widely divergent results [7]. Moreover, under certain conditions antioxidants may behave as prooxidants [5,8], which should be taken into account in more comprehensive antioxidant rankings.

The purpose of this work was to compare the protective effect of a range of antioxidants such as synthetic antioxidants, phenolic acids, flavonoids as well as other natural antioxidants against lipid peroxidation in two systems (egg yolk lipids and erythrocyte membrane lipids). The choice of antioxidants was a result of our previous participation in a project aimed at ranking of natural antioxidants as potential health-promoting agents [9]. We employed two systems for studying protection against lipid peroxidation: a homogenous, but biologically relevant system of egg yolk and a model membrane system (erythrocyte membranes, which are easy to prepare in a pure form). Then, five effective PBS-soluble antioxidants [2,5-dihydroxybenzoic acid (gentisic acid), (−)-epigallocatechin gallate (EGCG), (+)-catechin, gallic acid as well as ascorbic acid] were selected to study the protective effect of these compounds against oxidative stress (OS) on erythrocytes employing four different parameters. 2,2-Azobis (2-amidinopropane) dihydrochloride (AAPH) was chosen as a model oxidant inducing lipid peroxidation. This compound decomposes in a temperature-dependent manner generating free radicals; the half-life of AAPH is about 175 h at neutral pH, at the temperature of 37 °C, and the rate of free radical generation is 1.3 × 10^−6^ [AAPH]/s at these conditions [10]. Under aerobic conditions primary radicals are converted into peroxyl radicals, which induce oxidation of polyunsaturated lipids causing a chain reaction known as lipid peroxidation, and oxidation of other substrates [11,12,13].

This study demonstrates that for the same compounds, the relative antioxidant effects may differ even in simple model cell-free systems and prooxidant effects may be revealed at the level of a simple model cell such as the human erythrocyte even if they are not evident in other assays.

## 2. Results and Discussion

### 2.1. Inhibition of Lipid Peroxidation

We compared a range of synthetic and natural antioxidants with respect to inhibition of AAPH-induced lipid peroxidation in two systems: egg yolk suspension and erythrocyte membranes. Peroxidation rate was measured with BODIPY^®^ 581/591. This probe emits red fluorescence centered at 595 nm. Oxidation of the probe by peroxyl radicals is accompanied by decay of the red fluorescence and appearance of green fluorescence centered at about 520 nm. Decrease of the red fluorescence intensity, changes in the ratio of green and red fluorescence intensities or increase in the green fluorescence intensity have been used by various authors as indices of peroxidation [14,15,16]. In our hands, the rate of increase of the green fluorescence intensity was the most reproducible and reliable measure of the rate of peroxidation and was used in this study [16]. In egg yolk suspension, *tert*-butylhydroquinone (*t*-BHQ;) was the most efficient antioxidant, showing the lowest value of the concentration required for 50% inhibition of peroxidation (IC_50_).

Some natural antioxidants, including gallic acid, (−)-epicatechin gallate and melatonin showed comparable efficiency. Generally, hydrophilic antioxidants such as glutathione (GSH), cysteine, naringin, hesperetin and ascorbic acid exhibited lower efficiency. In another system, i.e., erythrocyte membranes (−)-epicatechin gallate was the most efficient, nevertheless the sequence of efficiency was different. While some antioxidants, such as (−)-epicatechin gallate or resveratrol had similar IC_50_ values in both system, the values for most compounds differed significantly in both systems, in most cases being higher for erythrocyte membranes (Table 1).

**Figure 1:**
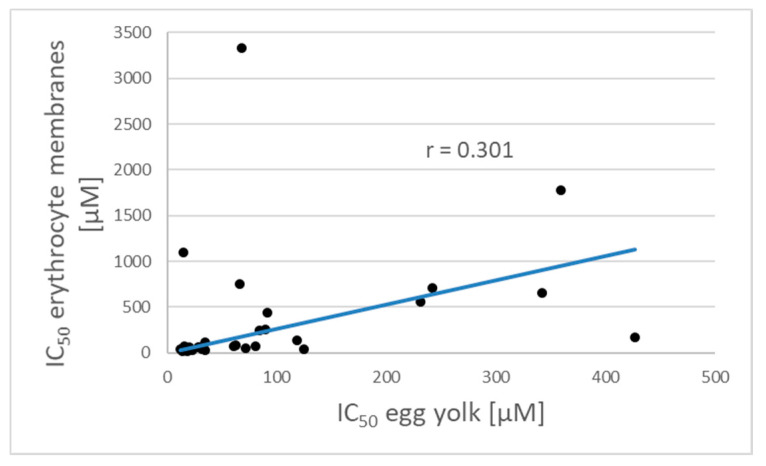
Correlation between IC_50_ values of antioxidants presented in Table 1 in two systems of lipid peroxidation: egg yolk suspension and erythrocyte membranes.

The Pearson linear correlation coefficient between IC_50_ values in both systems was only 0.30 (Figure 1). These differences may be due to the fact that in the more complex erythrocyte membrane systems some antioxidants may associate with membrane proteins and may be not, or only partly available for reactions with peroxide radicals in the lipid phase. The weight ratio of proteins to lipids is about 1:1 in erythrocyte membranes [17] and is significantly lower (about 0.5:1) in hen egg yolk [18], and protein composition differs considerably in both materials.

These results show that it may be misleading to extrapolate even results of evaluation of antioxidant potency to inhibit lipid peroxidation from one system to another.

### 2.2. Protection Against AAPH-Induced Hemolysis

For further experiments, five effective antioxidants soluble in PBS (to avoid the effects of organic solvent on the erythrocyte membrane), viz. (−)-epigallocatechin gallate, (+)-catechin, gallic acid, gentisic acid, and ascorbic acid were tested for their efficiency in preventing AAPH-induced oxidative damage on erythrocytes.

The hemolysis of erythrocytes has been extensively used as an ex vivo model in the study of ROS-induced disruption of cell membranes. AAPH is one of the most frequently studied compounds inducing oxidative hemolysis [19]. The mechanism of erythrocyte hemolysis induced by thermolysis of AAPH is not completely understood, but it has been correlated with lipid peroxidation and oxidation of membrane proteins. Accumulation of oxidized lipids around aggregated Band 3 protein most probably leads to the formation of hemolytic holes in the membrane [20].

Catechins (flavanols) are flavonoids that are found in black tea, green tea and other plant foods. They have been demonstrated to have numerous physiological effects, mostly dependent on their antioxidant action [21]. The antioxidant properties of polyphenols are mostly due to their redox properties, which let them act as reducing agents, hydrogen donors and singlet oxygen quenchers [22]. In contrast to GSH, polyphenols cannot be synthesized by humans, but are ingested in the diet; so, free radicals that are originated during body metabolism can be better neutralized by regular intake of a diet containing a high content of fruits and vegetables [23].

The best studied catechin is EGCG, the major polyphenol in green and black tea [24]. (+)-Catechin is mostly found in cacao and tea constituents, as well as in *Vitis vinifera* grapes, and is among the main polyphenols present in fruit wine [25]. Gallic acid (3,4,5-trihydroxybenzoic acid) is a natural phenolic antioxidant belonging to the most abundant phenolic antioxidants in wines and green tea [26]. Gallic acid is a phenolic acid, found in gallnuts, sumac, witch hazel, tea leaves, oak bark as well as other plants. This phytochemical is known for its antibacterial, anti-inflammatory, antimutagenic and chemopreventive properties. It is commonly used in the pharmaceutical industry as a standard for determination of the phenol content of various analytes by the Folin-Ciocalteau assay; results are usually reported in gallic acid equivalents [27,28]. Gentisic acid is a metabolite of acetylsalicylic acid, which shows a potent free radical scavenging activity with a minimal chelating effect. The antioxidant property of gentisic acid may partly account for the anti-atherogenic effects of aspirin [29]. It has been proposed that the antioxidant properties of gentisic acid are exerted by its phenoxyl group, leading to the formation of a phenoxyl radical. Ascorbate has been found to quench the phenoxyl radical of gentisic acid [30].

If the AAPH-induced oxidative hemolysis is mainly due to lipid peroxidation and membrane protein oxidation, membrane-interacting antioxidants should prevent hemolysis more effectively than those which do not interact with the membranes. All catechins are known to interact with the lipid bilayer, EGCG having higher affinity for membranes than non-esterified catechins [31]. However, AAPH is a water-soluble compounds and water-soluble antioxidants must be important in scavenging radicals generated by AAPH decomposition, while membrane-interacting antioxidants are crucial for inhibiting the chain process of lipid peroxidation [32].

**Figure 2:**
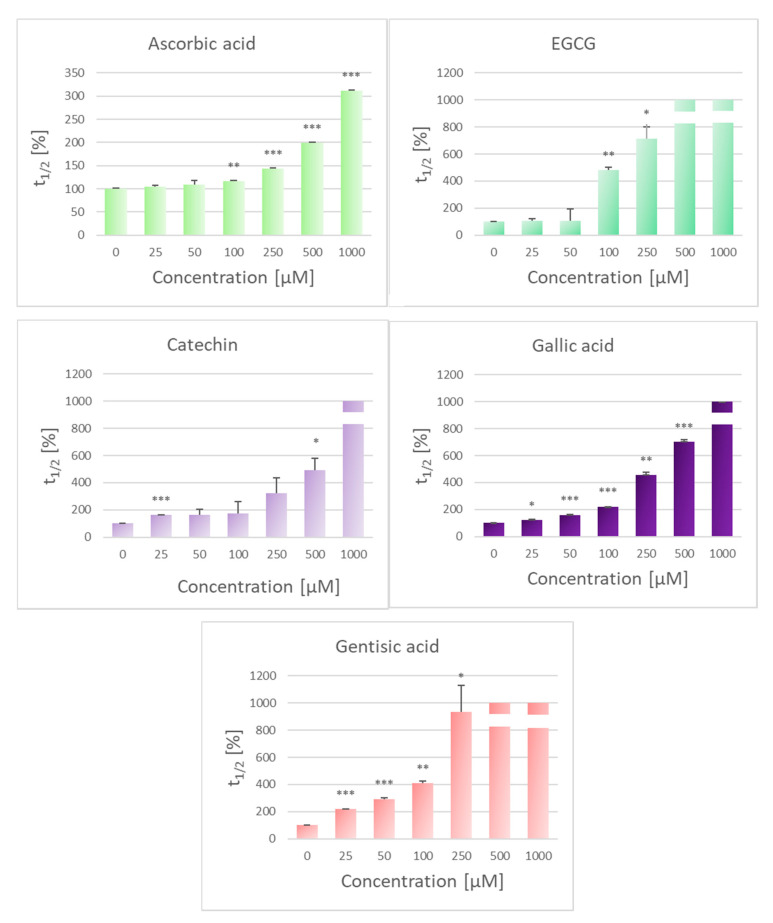
Effect of selected antioxidants on the AAPH-induced hemolysis of human erythrocytes. Hemolysis half-time expressed as percent of hemolysis half-time of “positive control” samples, containing no antioxidant (t_1/2_ = 96.3 ± 1.4 min), assumed as 100%. The turbidity of “negative control” samples, not added with AAPH, did not change during the incubation time. * *p* < 0.05, ** *p* < 0.01, *** *p* < 0.001 with respect to control (100%).

Most of the chosen antioxidants are partly associated with membranes, but also present in the aqueous medium while ascorbate is practically totally present in solution, so all of the antioxidants tested can interact with AAPH-generated radicals outside the cells. With respect to scavenging of the latter radicals, polyphenols can be expected to be more reactive than ascorbic acid, due to the higher number of reactive hydroxyl groups [33]. Indeed, although all antioxidants studied were effective, the highest concentrations of membrane-interacting antioxidants prolonged the time necessary to obtain a 50% decrease in the turbidity of erythrocyte suspensions to more than 1000%, while 1 mM ascorbic acid increased the relative hemolysis time to only about 300% (Figure 2). The efficiency of a hydrophilic antioxidant, ascorbic acid, may be contributed by its property of regeneration of radicals of other antioxidants formed in the process of inhibition of lipid peroxidation [34,35].

### 2.3. Attenuation of ROS Level

**Figure 3:**
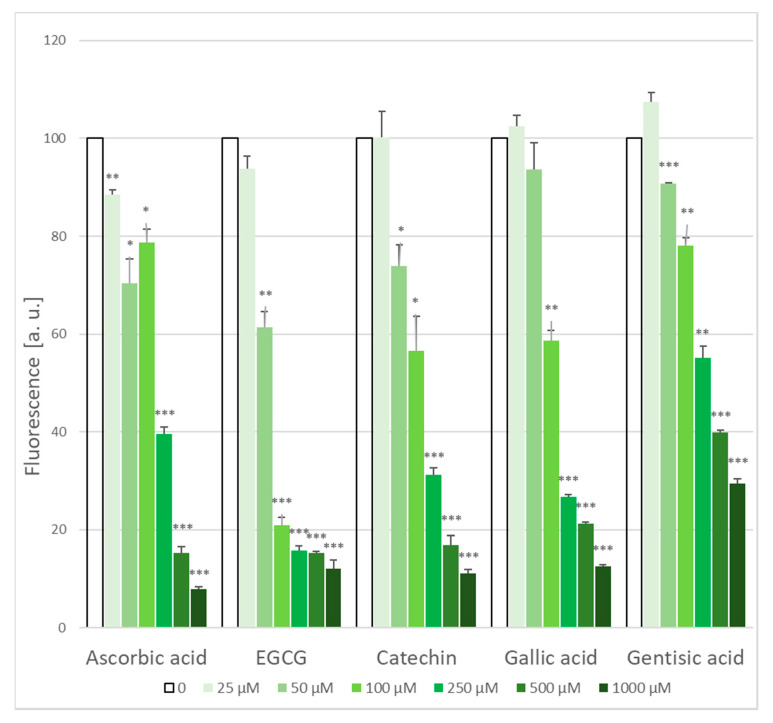
Attenuation of AAPH-generated intracellular ROS level by chosen antioxidants. The ROS level in the absence of any additive assumed as 100%. Endogenous ROS level in the absence of AAPH: 8.5 ± 0.3% of that found with AAPH. * *p* < 0.05, ** *p* < 0.01, *** *p* < 0.001 with respect to control (100%).

Exposure to AAPH induced an abundant generation of ROS reacting with H_2_DCF-DA. All the antioxidants used dose-dependently decreased the ROS level inside erythrocytes (Figure 3). The IC_50_ values of the antioxidants tested for the inhibition of ROS formation in erythrocytes are given in Table 2. Interestingly, EGCG proved to be the most effective antioxidant in this test, catechin and gallic acid showed similar effectivity while gentisic acid was the least effective, in spite of its structural similarity to gallic acid.

### 2.4. Protection against Hemoglobin Oxidation

**Figure 4:**
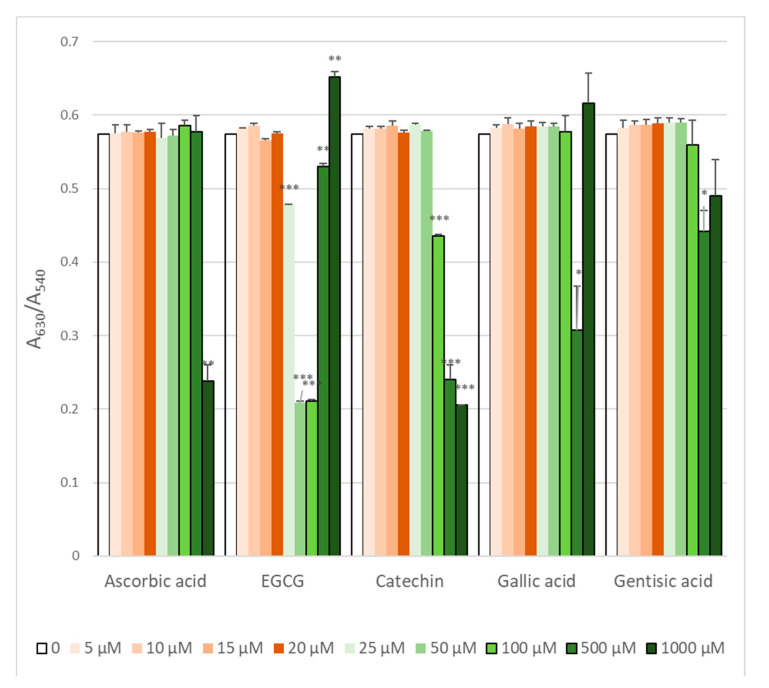
Prevention of AAPH-induced hemoglobin oxidation by selected antioxidants. * *p* < 0.05, ** *p* < 0.01, *** *p* < 0.001 with respect to control.

**Figure 5:**
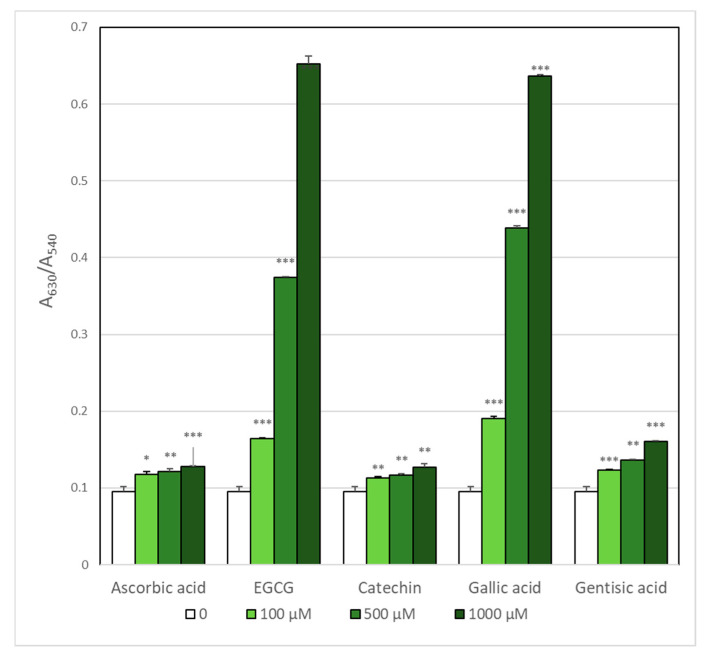
Oxidation of hemoglobin by selected antioxidants in the absence of AAPH. * *p* < 0.05, ** *p* < 0.01, *** *p* < 0.001 with respect to control.

ROS generated by AAPH-induced oxidation of intracellular hemoglobin. As hemoglobin is present at very high concentration inside erythrocytes (over 30% by weight), it is obvious that only high concentrations of antioxidants were effective in preventing hemoglobin oxidation (Figure 4). However, EGCG and gallic acid were effective only in some concentration range while the protective effect disappeared with a further increase in the antioxidant concentration. As this effect was reproducible, we checked if the antioxidants studied oxidized hemoglobin themselves. The results obtained for the highest antioxidant concentration showed that, indeed, EGCG and gallic acid were able to oxidize hemoglobin (Figure 5), which explains the strange dose dependence observed.

Previously, oxidation of hemoglobin to methemoglobin by black and green tea extracts, containing EGCG, was noted [35]. Both gallic acid and EGCG contain the trihydroxybenzoic acid residue, which might be responsible for their prooxidative effect on hemoglobin.

### 2.5. Glutathione Loss

**Figure 6:**
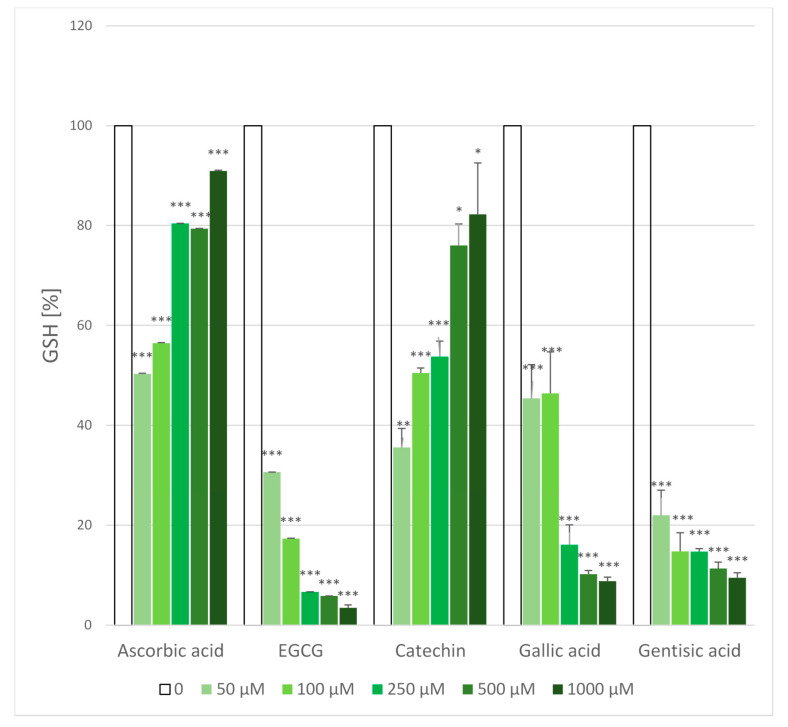
Effect of antioxidants on the AAPH-induced loss of erythrocyte glutathione (GSH). GSH level in AAPH-treated erythrocytes was assumed as 100%. * *p* < 0.05, ** *p* < 0.01, *** *p* < 0.001 with respect to control.

**Figure 7:**
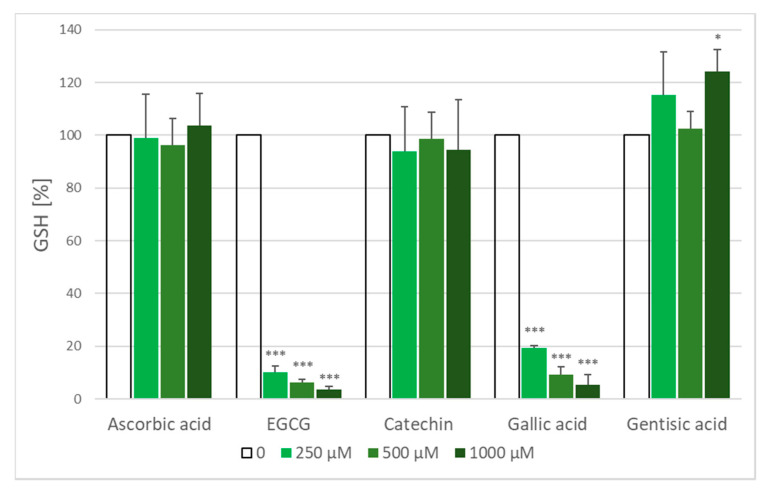
Effect of antioxidants on the GSH level in erythrocytes in the absence of AAPH. The level of GSH in control erythrocytes was assumed as 100%. * *p* < 0.05, *** *p* < 0.001 with respect to control.

Treatment of erythrocyte suspension with AAPH solution for 1 h resulted in a decrease of erythrocyte glutathione concentration from a value of 1875 ± 164 µM to 865 ± 233 µM i.e., by 54%. Ascorbic acid and catechin dose-dependently protected against GSH loss. On the contrary, EGCG, gallic acid and gentisic acid significantly augmented GSH loss (Figure 6). When erythrocytes were incubated with antioxidants under the same conditions but without AAPH, ascorbic acid, catechin and gentisic acid did not decrease the GSH level, while higher concentrations of EGCG and gallic acid produced a considerable GSH loss (Figure 7). Gallic acid has been reported to oxidize glutathione in erythrocytes [19] and EGCG-rich extracts of black and green tea were found to decrease the level of erythrocyte GSH [35]. Thus, the data for EGCG and gallic acid are understandable (GSH loss induced by these compound alone is superposed on the AAPH-induced loss).

The results for gentisic acid are more difficult to explain. It can be postulated that reactive species formed by interaction of AAPH with gentisic acid may exhibit prooxidative properties not shown by native gentisic acid. We and others have shown such that other antioxidants, melatonin and resveratrol showed prooxidant effects on glyceraldehyde 3-phosphate dehydrogenase when applied together with nitric oxide; this effect was due to formation of their reactive derivatives, viz. phenoxyl radical in the case of resveratrol [36,37]. As a phenoxyl radical is formed also during oxidation of gentisic acid [30], the mechanism of the prooxidant action of gentisic acid may be similar.

An obvious outcome of this study is the limited correlation or lack of correlation between results of various assays of antioxidant efficiency of the same compounds. EGCG and gallic acid, which are good antioxidants in the test of inhibition of lipid peroxidation and hemolysis, promoted hemoglobin oxidation and glutathione loss. However, another aspect of the prooxidant effects of some antioxidants is their physiological relevance. It should be noted that the latter effects were observed under model experimental conditions, with antioxidant concentrations not attainable in vivo and under oxygen concentrations much higher than those prevailing inside the body. Under conditions which can be physiologically achieved, the prooxidants effects of these compounds are most probably of negligible significance. From that point of view, limited bioavailability of many food antioxidants, may be not necessarily their disadvantage, preventing their undesired reactions in the body, and could be even an evolutionary adaptation.

## 3. Materials

### Chemicals and Equipment

Dimethyl sulfoxide (DMSO; purity: ≥99.9% sterile filtered) produced by BioShop Canada Inc. (Burlington, ON, Canada) was purchased from Lab Empire (Rzeszów, Poland). 2,2-Azobis(2-amidinopropane) dihydrochloride (AAPH) was purchased from Polysciences (Warrington, PA, USA). A stock solution of AAPH was freshly prepared in PBS before each experiment. 2′,7′-dichlorodihydrofluorescein diacetate (H_2_DCF-DA), Rutin trihydrate and Lipid Peroxidation Sensor (4,4-difluoro-5-(4-phenyl-1,3-butadienyl)-4-bora-3a,4a-diaza-s-indacene-3-un-decanoic acid; C11-BODIPY^®^ 581/591) were purchased from Thermo Fisher Scientific (Warsaw, Poland). Daidzein was purchased from Santa Cruz Biotechnology (Dallas, TX, USA).

All other reagents, if not mentioned otherwise, were purchased from Sigma (Poznan, Poland) and were of analytical grade. Distilled water was purified using a Milli-Q system (Millipore, Bedford, MA, USA). Fluorometric and absorptiometric measurements were done in an Infinite 200 PRO multimode reader (C11-BODIPY fluorescent microplate assay) or a Spark multimode microplate reader (Tecan Group Ltd., Männedorf, Switzerland). All measurements were performed in triplicate and repeated at least three times. Other reagents were dissolved in PBS or DMSO. Minimal amounts of the solvents present in the samples had a small effect on the protection (up to a few %). The effect of DMSO was subtracted from the effects of substances introduced in this solvent.

## 4. Methods

### 4.1. Experiments in a Cell-Free System

#### Lipid Peroxidation

Forty μL of 10% egg yolk suspension in PBS (1 mL of egg yolk suspended with vigorous vortexing in PBS) was treated with 50 mM AAPH (final concentration) in the presence of selected antioxidants. The concentration range of these compounds was 0.01–5 mM. The intensely fluorescent BODIPY^®^581/1591 (4,4-difluoro-5-(4-phenyl-1,3-butadienyl)-4-bora-3a,4a-diaza-s-indacene-3-un-decanoic acid; C11-BODIPY) fluorophore was used for monitoring lipid peroxidation. BODIPY^®^ 581/591 undecanoic acid (5 μL of 0.1 mM solution in DMSO) was added to each well of a black 96-well plate. The final volume of a sample was 200 μL. The kinetic measurement of fluorescence increase at 526 nm was carried using the excitation wavelength of 485 nm at 37 °C for 150 min and the rate of fluorescence increase was measured.

Percent protection against lipid peroxidation by selected antioxidants was calculated as:

% Protection = 100% [1 − (A_n_ − A_c_)/(A_o_ − A_c_)]

where A_o_—fluorescence of sample incubated with AAPH; A_n_—fluorescence of a sample containing a protective agent; A_c_—fluorescence of a sample not treated with an oxidizing agent.

### 4.2. Experiment with Erythrocytes

#### 4.2.1. Ethical Approval

The study was approved by the Bioethics Committee of the University of Lodz (Permit No. KBBN-UŁ/I/3/2013).

#### 4.2.2. Preparation of Erythrocytes

Eight milliliters of peripheral blood from healthy donors (lab volunteers) were collected in EDTA tubes and used within the day of its collection. Erythrocytes were isolated by centrifugation for 10 min at 3000× *g* at 4 °C. The plasma and buffy coat were removed by aspiration. The red blood cells (RBCs) were washed four times with ice-cold PBS. Washed RBCs were suspended at various hematocrit from 4% to 60%.

#### 4.2.3. Preparation of Erythrocyte Ghosts

Erythrocyte ghosts were prepared from washed erythrocytes according to the method of Dodge et al. [17] with some modifications. Briefly, after incubation, erythrocytes were hemolyzed on ice with 20 volumes of 20 mM phosphate buffer, pH 7.4, containing 1 mM ethylenediaminetetraacetic acid (EDTA) and centrifuged at 4 °C at 20,000× *g* for 20 min. The ghosts were resuspended in ice-cold 10 mM and then 5 mM phosphate buffer, pH 7.4 containing 1 mM EDTA, centrifuged, and this process was continued until the ghosts were free from residual hemoglobin. Finally, the erythrocyte ghosts were resuspended in 20 mM phosphate buffer, pH 7.4. The protein concentration was estimated by the method of Lowry et al. [38].

#### 4.2.4. Estimation of the Protective Effects of Selected Compounds on Erythrocyte Membrane Lipid Peroxidation

BODIPY^®^ 581/591 undecanoic acid was dissolved in DMSO by adding 475 μL DMSO to 25 μL 2 mM BODIPY^®^ 581/591 stock solution (dissolved also DMSO). First, PBS was pipetted so that the volume of each well of a black 96-well plate was 200 μL. Next, 40 μL aliquots of membrane suspensions were pipetted into the 96-well black plate, followed by selected compounds in a range of concentrations 10–1000 µM (10 mM GSH, *N*-acetyl-l-cysteine, l-ascorbic acid stock solutions in PBS or 10 mM BHA, mangiferin, naringenin, hesperetin, rutin trihydrate, naringin, *p*-coumaric acid, hesperidin, ferulic acid, chlorogenic acid, Trolox stock solutions in DMSO and then diluted with PBS to obtain concentrations of 5 or 1 mM), 5 μL of BODIPY^®^ 581/591 (0.1 mM stock solution) as well as at the end 100 μL AAPH (100 mM stock solution). The fluorescence (480 nm/524 nm) was measured every 2 min for 150 min. Percent protection against lipid peroxidation by selected antioxidants was calculated as above.

#### 4.2.5. The Assay of AAPH-Induced Hemolysis

The inhibition of free radical-induced RBCs hemolysis was performed by a modification of a previously published method [39], in which hemolysis was monitored turbidimetrically. Hemolysis was induced by thermal decomposition of AAPH. The protective effect of selected antioxidants against AAPH-induced hemolysis was measured only for compounds dissolved in PBS. The RBCs suspension was added with appropriate antioxidant solution to a final concentration in the range of 25–1000 µM and incubated with shaking in the presence/absence of 50 mM AAPH, as optimal concentration to induce hemolysis at 37 °C. The absorbance (700 nm) was measured every 1 h for 12 h using the Tecan Infinite 200 PRO multimode reader. All measurements were performed in triplicate and repeated at least three times. For all determinations, hemolysis time and percentage of hemolysis time with respect to control erythrocytes were calculated as 100% × [time (min) for test compound/mean time (min) for control sample containing RBCs and AAPH only].

#### 4.2.6. Determination of Intracellular ROS Generation

2′,7′-Dichlorodihydrofluorescein diacetate (H_2_DCF-DA, also known as 2′,7′-dichlorofluorescin diacetate) is a chemically reduced form of 2′,7′-dichlorofluorescein diacetate used as an indicator for ROS in cells. Upon cleavage of the acetate groups by intracellular esterases as well as oxidation, the nonfluorescent H_2_DCF-DA is converted to the highly fluorescent 2′,7′-dichlorofluorescein (DCF) [39,40]. The indicator H_2_DCF-DA (10 mM stock solution in DMSO) was added to the erythrocyte suspension (10% final hematocrit) to a final concentration of 10 µM of with subsequent incubation at 37 °C for 30 min. Then, samples were washed 2 times with ice cold PBS by centrifugation at 4000× *g* for 5 min and supernatant was discarded to remove excess H_2_DCF-DA. Afterwards, 10 μL of suspension of H_2_DCF-DA-loaded erythrocytes (hematocrit of 10%) were pipetted into wells of a 96-well black plate containing appropriate amount of PBS to provide final volume of 200 μL, followed by addition of an appropriate antioxidant solution in PBS to obtain concentrations in the range of 25–1000 µM. AAPH (50 mM final concentration) was then added with good mixing. The fluorescence (485 nm/529 nm) was measured every 2 min for 120 min and sum of fluorescence values obtained in successive measurements was calculated. From these sums of fluorescence values, per cent inhibition of ROS formation and IC_50_ values (antioxidant concentration inhibiting ROS formation by 50%) were calculated.

#### 4.2.7. Hemoglobin Oxidation

Aliquots of RBC suspensions in PBS (hematocrit of 10%) were added with appropriate antioxidant (final concentration range of 5–1000 μM) and AAPH (final concentration of 50 mM), and incubated with shaking at 37 °C for 5 h. A set of control samples contained antioxidants but no AAPH. After incubation, 1.5 mL distilled water was added to the 0.4 mL samples, the samples were mixed and centrifuged (3 min, 8000× *g*). Next, 200 µL of supernatants were taken and added to a transparent 96-well plate. The absorbance was measured at the wavelengths of 540 and 630 nm. The absorbance ratio A_630_/A_540_ was calculated as a measure of hemoglobin oxidation.

#### 4.2.8. Glutathione Content

Suspensions of erythrocytes (RBCs) in PBS (final hematocrit of 2%) were treated with different concentration of selected antioxidants (5 mM stock solution) and/or AAPH (100 mM stock solution) and incubated for 1 h at 37 °C with continuous shaking. Then, all samples were centrifuged at 10,000× *g* for 3 min and the supernatants were discarded. Samples were washed with 300 µL of cold 1 × PBS (pH = 7.2). The erythrocyte pellet was precipitated with cold 100 µL RQB-TCA buffer (20 mM HCl, 5 mM diethylenetriaminepentaacetic acid, 10 mM ascorbic acid, 5% trichloroacetic acid), kept on ice for 10 min, centrifuged (13,000× *g*, 2 °C, 5 min) and the supernatant was taken for the GSH assay [41]. For GSH determination, 5 µL of deproteinized supernatant diluted by adding 25 µL RQB-TCA were put on two wells (denoted ‘−’ and ‘+’) of a 96-well black plate. The sample ‘−’ was added with 4 µL of 7.5 mM N-ethylmaleimide in RQB-TCA, both samples added with 40 µL of 1 M potassium phosphate (pH = 7.0), mixed and incubated at room temperature for 5 min. Then 160 µL of 0.1 M potassium phosphate buffer (pH = 7.0), was added, followed by 25 µL of 0.5% *o*-phtalaldehyde in methanol and the plate was shaken (1 min). After 30-min incubation (room temperature) the fluorescence was read at 355 nm/460 nm. The value obtained for the ‘−’ sample was subtracted from that obtained for the ‘+’ value and GSH concentration was read from a calibration curve obtained with glutathione as a standard.

#### 4.2.9. Statistical Analysis

Statistical significance of differences was evaluated using paired Student’s “t” test. Statistical analysis of the data was performed using the STATISTICA software package (version 12, StatSoft Inc. 2014, Tulsa, OK, USA, www.statsoft.com).

## 5. Conclusions

Results of this study demonstrate a limited correlation between various assays of antioxidant activity. Some assays reveal prooxidant effect of antioxidants which are not evident in other systems. Thus, rankings of antioxidants should not be based on a single parameter, but take into account their behavior in different systems.

## Figures and Tables

**Table 1:** Inhibition of BODIPY^®^ 581/591 oxidation by synthetic and natural antioxidants.

Compound	IC_50_ Egg Yolk [µM]	IC_50_ Erythrocyte Membranes [µM]
Synthetic antioxidants		
*t*-BHQ	12.04 ± 3.55	37.57 ± 1.91 ***
Pyrogallol	60.79 ± 14.17	70.80 ± 6.46
Trolox	62.78 ± 2.90	87.46 ± 7.38 **
BHA	71.42 ± 15.54	50.04 ± 5.26 *
*N*-Acetyl-l-cysteine	231.50 ± 2.21	554.99 ± 60.34 ***
Phenolic acids		
Gallic acid	15.33 ± 6.81	72.35 ± 4.09 ***
Caffeic acid	15.45 ± 0.33	34.92 ± 0.40 ***
Propyl gallate	19.60 ± 3.04	65.32 ± 3.27 ***
Chlorogenic acid	22.92 ± 1.91	33.33 ± 1.22 ***
2,5-Dihydroxybenzoic acid (Gentisic acid)	29.38 ± 6.22	56.52 ± 0.60 ***
Vanillic acid	34.67 ± 6.34	114.72 ± 17.69 ***
Ferulic acid	80.80 ± 2.33	74.87 ± 1.12 **
*p*-Coumaric acid	89.28 ± 0.12	256.39 ± 31.65 ***
Sinapic acid	124.71 ± 4.67	40.83 ± 1.6 ***
Flavonoids		
(−)-Epicatechin gallate	13.68 ± 0.51	14.46 ± 0.39
(−)-Epicatechin	16.45 ± 1.22	27.06 ± 1.04 ***
Quercetin	17.89 ± 0.83	27.67 ± 1.29 ***
(−)-Epigallocatechin gallate	18.16 ± 0.55	22.64 ± 0.03 ***
(+)-Catechin	21.26 ± 0.67	30.51 ± 1.1 ***
Mangiferin	21.98 ± 6.32	35.95 ± 3.59 *
(−)-Epigallocatechin	28.35 ± 0.15	61.34 ± 2.48 ***
Rutin trihydrate	34.40 ± 0.63	27.94 ± 1.12 ***
Daidzein	67.37 ± 6.36	3331.1 ± 429.76 ***
Hesperidin	84.41 ± 7.23	242.86 ± 3.72 ***
Naringenin	91.67 ± 5.40	441.84 ± 30.72 ***
Naringin	359.11 ± 59.28	1782.29 ± 76.34 ***
Hesperetin	426.96 ± 18.13	165.58 ± 6.15 ***
Other natural antioxidants		
Melatonin	14.37 ± 4.29	1098.96 ± 65.19 ***
Resveratrol	31.38 ± 2.79	35.57 ± 3.59
β-Carotene	66.19 ± 10.40	748.75 ± 20.35 ***
l-Ascorbic acid	118.71 ± 5.38	138.19 ± 7.24 *
l-Cysteine	241.95 ± 52.86	705.92 ± 32.68 ***
Glutathione	341.85 ± 7.09	655.90 ± 41.89 ***

Antioxidant concentrations providing 50% inhibition of the probe oxidation (IC_50_) are presented (mean ± SD, *n* > 3). *t*-BHQ, *tert*-butylhydroquinone; BHA, butylated hydroxyanisole; * *p* < 0.05, ** *p* < 0.01, *** *p* < 0.001 with respect to the value obtained for egg yolk suspension.

**Table 2:** IC_50_ values of chosen antioxidants for inhibition of ROS formation in erythrocytes. Mean ± SD, *n* ≥ 3.

Compound	IC_50_ [µM]
Ascorbic acid	209.7 ± 35.2
EGCG	64.0 ± 18.2
Catechin	138.5 ± 29.4
Gallic acid	140.3 ± 20.0
Gentisic acid	333.3 ± 46.2

## References

[1] Kampa R.P., Kicinska A., Jarmuszkiewicz W., Pasikowska-Piwko M., Dolegowska B., Debowska R., Szewczyk A., Bednarczyk P. (2019). Naringenin as an opener of mitochondrial potassium channels in dermal fibroblasts. Exp. Dermatol..

[2] Lanigan R.S., Yamarik T.A. (2002). Final report on the safety assessment of BHT (1). Int. J. Toxicol..

[3] Ito N., Fukushima S., Tsuda H. (1985). Carcinogenicity and modification of the carcinogenic response by BHA, BHT, and other antioxidants. Crit. Rev. Toxicol..

[4] Poulsen E. (1991). Safety evaluation of substances consumed as technical ingredients (food additives). Food Addit. Contam..

[5] Carocho M., Ferreira I.C. (2013). A review on antioxidants, prooxidants and related controversy: Natural and synthetic compounds, screening and analysis methodologies and future perspectives. Food Chem. Toxicol..

[6] Gilgun-Sherki Y., Melamed E., Offen D. (2001). Oxidative stress induced neurodegenerative diseases: The need for antioxidants that penetrate the blood brain barrier. Neuropharmacology.

[7] Cui L., Decker E.A. (2016). Phospholipids in foods: Prooxidants or antioxidants?. J. Sci. Food Agric..

[8] Villanueva C., Kross R.D. (2012). Antioxidant-induced stress. Int. J. Mol. Sci..

[9] Grzesik M., Bartosz G., Stefaniuk I., Pichla M., Namieśnik J., Sadowska-Bartosz I. (2019). Dietary antioxidants as a source of hydrogen peroxide. Food Chem..

[10] Niki E. (1990). Free radical initiators as source of water- or lipid-soluble peroxyl radicals. Methods Enzymol..

[11] Zou C.G., Agar N.S., Jones G.L. (2001). Oxidative insult to human red blood cells induced by free radical initiator AAPH and its inhibition by a commercial antioxidant mixture. Life Sci..

[12] Ji J.A., Zhang B., Cheng W., Wang Y.J. (2009). Methionine, tryptophan, and histidine oxidation in a model protein, PTH: Mechanisms and stabilization. J. Pharm. Sci..

[13] Wu W., Zhang C., Kong X., Hua Y. (2009). Oxidative modification of soy protein by peroxyl radicals. Food Chem..

[14] Drummen G.P., van Liebergen L.C., Op den Kamp J.A., Post J.A. (2002). C11-BODIPY(581/591), an oxidation-sensitive fluorescent lipid peroxidation probe: (Micro)Spectroscopic characterization and validation of methodology. Free Radic. Biol. Med..

[15] Carlsen C.U., Kurtmann L., Brüggemann D.A., Hoff S., Risbo J., Skibsted L.H. (2009). Investigation of oxidation in freeze-dried membranes using the fluorescent probe C11-BODIPY(581/591). Cryobiology.

[16] Zhu M., Qin Z.J., Hu D., Munishkina L.A., Fink A.L. (2006). Alpha-synuclein can function as an antioxidant preventing oxidation of unsaturated lipid in vesicles. Biochemistry.

[17] Dodge J.T., Mitchell C., Hanahan D.J. (1963). The preparation and chemical characteristics of hemoglobin-free ghosts of human erythrocytes. Arch. Biochem. Biophys..

[18] Sugino H., Nitoda T., Junoja L.R., Yamamoto T., Juneja L.R., Hatta H., Kim M. (1997). General chemical composition of hen eggs. Hen Eggs, Their Basic and Applied Science.

[19] Ximenes V.F., Lopes M.G., Petrônio M.S., Regasini L.O., Silva D.H., da Fonseca L.M. (2010). Inhibitory effect of gallic acid and its esters on 2,2′-azobis(2-amidinopropane)hydrochloride (AAPH)-induced hemolysis and depletion of intracellular glutathione in erythrocytes. J. Agric. Food Chem..

[20] Sato Y., Kamo S., Takahashi T., Suzuki Y. (1995). Mechanism of free radical-induced hemolysis of human erythrocytes: Hemolysis by water-soluble radical initiator. Biochemistry.

[21] Kalender Y., Kaya S., Durak D., Uzun F.G., Demir F. (2012). Protective effects of catechin and quercetin on antioxidant status, lipid peroxidation and testis-histoarchitecture induced by chlorpyrifos in male rats. Environ. Toxicol. Pharmacol..

[22] Lima G.P.P., Vianello F., Corrêa C.R., da Silva Campos R.A., Borguini M.G. (2014). Polyphenols in fruits and vegetables and its effect on human health. Food Nutr. Sci..

[23] Milella L., Caruso M., Galgano F., Favati F., Padula M.C., Martelli G. (2011). Role of the cultivar in choosing Clementine fruits with a high level of health-promoting compounds. J. Agric. Food Chem..

[24] Fujiki H. (2005). Green tea: Health benefits as cancer preventive for humans. Chem. Rec..

[25] Gadkari P.V., Balaraman M. (2015). Catechins: Sources, extraction and encapsulation: A review. Food Bioprod. Proc..

[26] Reckziegel P., Dias V.T., Benvegnú D.M., Boufleur N., Barcelos R.C.S., Segat H.J., Pase C.S., Dos Santos C.M.M., Flores É.M.M., Bürger M.E. (2016). Antioxidant protection of gallic acid against toxicity induced by Pb in blood, liver and kidney of rats. Toxicol. Rep..

[27] Choubey S., Goyal S., Varughese L.R., Kumar V., Sharma A.K., Beniwal V. (2018). Probing Gallic Acid for Its Broad Spectrum Applications. Mini Rev. Med. Chem..

[28] Kahkeshani N., Farzaei F., Fotouhi M., Alavi S.S., Bahramsoltani R., Naseri R., Momtaz S., Abbasabadi Z., Rahimi R., Farzaei M.H. (2019). Pharmacological effects of gallic acid in health and diseases: A mechanistic review. Iran. J. Basic Med. Sci..

[29] Ashidate K., Kawamura M., Mimura D., Tohda H., Miyazaki S., Teramoto T., Yamamoto Y., Hirata Y. (2005). Gentisic acid, an aspirin metabolite, inhibits oxidation of low-density lipoprotein and the formation of cholesterol ester hydroperoxides in human plasma. Eur. J. Pharmacol..

[30] Joshi R., Gangabhagirathi R., Venu S., Adhikari S., Mukherjee T. (2012). Antioxidant activity and free radical scavenging reactions of gentisic acid: In-vitro and pulse radiolysis studies. Free Radic. Res..

[31] Nakayama T., Hashimoto T., Kajiya K., Kumazawa S. (2000). Affinity of polyphenols for lipid bilayers. Biofactors.

[32] Minnelli C., Galeazzi R., Laudadio E., Amici A., Rusciano D., Armeni T., Cantarini M., Stipa P., Mobbili G. (2020). Monoalkylated Epigallocatechin-3-gallate (C18-EGCG) as Novel Lipophilic EGCG Derivative: Characterization and Antioxidant Evaluation. Antioxidants.

[33] Abramovic H., Grobin B., Poklar Ulrih N., Blaˇz Cigi B. (2018). Relevance and Standardization of In Vitro Antioxidant Assays: ABTS, DPPH, and Folin–Ciocalteu. J. Chem..

[34] McCay P.B. (1985). Vitamin E: Interactions with free radicals and ascorbate. Annu. Rev. Nutr..

[35] Ko C.H., Li K., Ng P.C., Fung K.P., Li C.L., Wong R.P.-O., Chui K.M., Gu G.J.-S., Yung E., Wang C.C. (2006). Pro-oxidative effects of tea and polyphenols, epigallocatechin-3-gallate and epigallocatechin, on G6PD-deficient erythrocytes in vitro. Int. J. Mol. Med..

[36] Rodacka A., Strumillo J., Serafin E., Puchala M. (2014). Effect of Resveratrol and Tiron on the Inactivation of Glyceraldehyde-3-phosphate Dehydrogenase Induced by Superoxide Anion Radical. Curr. Med. Chem..

[37] Strumillo J., Nowak K.E., Krokosz A., Rodacka A., Puchala M., Bartosz G. (2018). The role of resveratrol and melatonin in the nitric oxide and its oxidation products mediated functional and structural modifications of two glycolytic enzymes: GAPDH and LDH. Biochim. Biophys. Acta Gen. Subj..

[38] Lowry O.H., Rosebrough N.J., Farr A.L., Randall R.J. (1951). Protein measurement with the Folin phenol reagent. J. Biol. Chem..

[39] Wang J., Sun B., Cao Y., Tian Y. (2009). Protection of wheat bran feruloyl oligosaccharides against free radical-induced oxidative damage in normal human erythrocytes. Food Chem. Toxicol..

[40] Wang G., Lei Z., Zhong Q., Wu W., Zhang H., Min T., Wu H., Lai F. (2017). Enrichment of caffeic acid in peanut sprouts and evaluation of its in vitro effectiveness against oxidative stress-induced erythrocyte hemolysis. Food Chem..

[41] Senft A., Dalton T., Shertzer H. (2000). Determining glutathione and glutathione disulfide using the fluorescence probe *o*-phthalaldehyde. Anal. Biochem..

